# A Modified Technique for All-Inside Anterior Cruciate Ligament Reconstruction (ACLR): True Femoral Socket

**DOI:** 10.1016/j.eats.2023.07.025

**Published:** 2023-10-23

**Authors:** Wenbo Yang, Zhen Huang, Zengwu Shao, Hong Wang, Wei Huang

**Affiliations:** a Department of Orthopaedics, Union Hospital, Tongji Medical College, Huazhong University of Science and Technology, Wuhan, China; b Department of Orthopaedics, Enshi huiyi Hospital of Rheumatic, Enshi, China

## Abstract

Anterior cruciate ligament reconstruction (ACLR) performed via arthroscopy is the primary treatment for anterior cruciate ligament injury. In traditional ACLR, the surgeon must create bone tunnels in both the femur and tibia, which increases the risk of bleeding and pain. The advent of all-inside technology has introduced the concept of bone sockets. However, the femoral socket created by the traditional all-inside technique is not a true femoral socket since the tunnel ends are still connected to achieve suspensory fixation. We are dedicated to achieving a true femoral socket in the all-inside ACLR technique. The AperFix Implant fixation system offers the potential for a genuine femoral socket by securely holding the ligaments in place through compression fixation. In this report, we present an all-inside ACLR using the AperFix Implant fixation system, which allows for a single exit of the femur side tunnel. This technique effectively reduces “windshield wiper” effect, “bungee cord” effect, as well as surgical time and minimizes the risk of bleeding, pain, and local microfractures.

Anterior cruciate ligament (ACL) injury is a common knee injury. Because the ACL is closely related to the mechanical structure of the knee joint, reconstructive surgery is the most common treatment for the current rupture of the ACL. The purpose of ACLR is to replace the anterior cruciate ligament with a graft to maintain knee stability. When autologous tendon is used for ACLR, tendon diameter, fixation mode, and bone tunnel morphology are the key factors affecting the effect of ACLR. In conventional ACLR, tendons are commonly fixed by a cortical suspensory fixation button at the anterior cruciate ligament on the femur side and by compression screw suspension at the tibia side.^1^ The existence of bone tunnel and poor healing of tendon and bone often lead to “windshield-wiper” and “bungee cord”, which may lead to bone tunnel expansion and anterior cruciate ligament reconstruction failure in some cases.^2^ Therefore, it is necessary to improve the traditional ACLR technology. All-inside technology is a new ACLR technology developed in recent years.^3^^,^^4^ The all-inside technique eliminates the need for traditional bone tunneling and screw fixation by establishing bone sockets as a channel for delivery of graft and using suspensory fixation for any type of ACLR. However, because the cortical suspensory fixation button is still required, the femur tunnel, as well as the tibia tunnel, actually still has connected “exits”.^5^ Fixation only at ends leaves the risk of “windshield wiper” effect, “bungee cord” effect, and even localized microfractures still present. Without affecting the stability of the graft, if the "femoral socket" can be true aperture fixation, the convenience of ACLR will be greatly improved, and the surgical effect will be further improved. On the basis of this requirement, we note that AperFix Implant fixation system.^6^ The AperFix Implant fixation system was designed to achieve circumferential graft compression in situ by incorporating a simple mechanism to complete the bone end fixation of the graft. This technique allows the true femoral socket fixation in femur to be implemented in ACLR. Therefore, we applied this technique in conjunction with all-inside technique for ACLR, with the aim to improve on current All-inside technique to achieve better clinical results. A diagram of our improved technique is shown in Figure 1.

**Fig 1:**
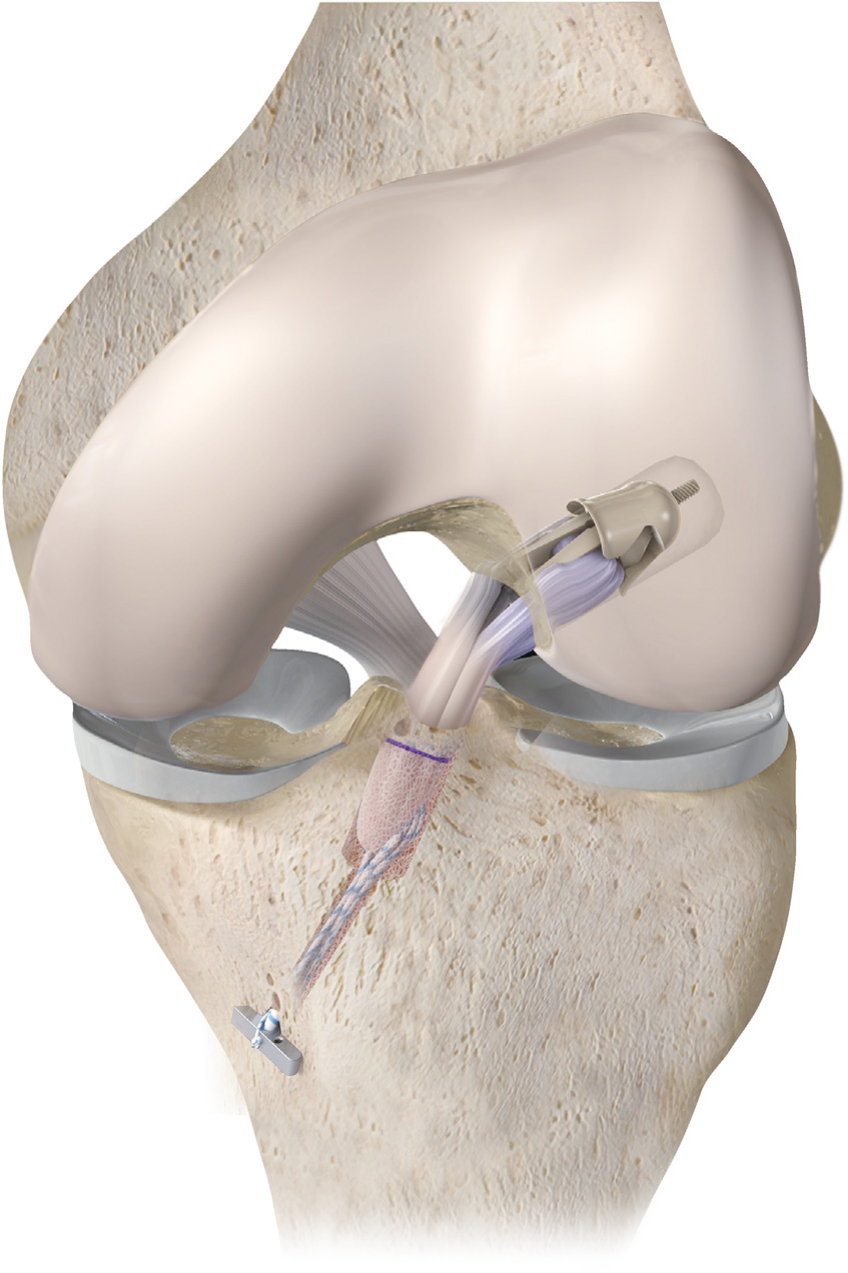
An animated scheme of our improved technology.

## Surgical Technique

### Technique Overview, Patient Evaluation, Imaging, and Indications

**Fig 2:**
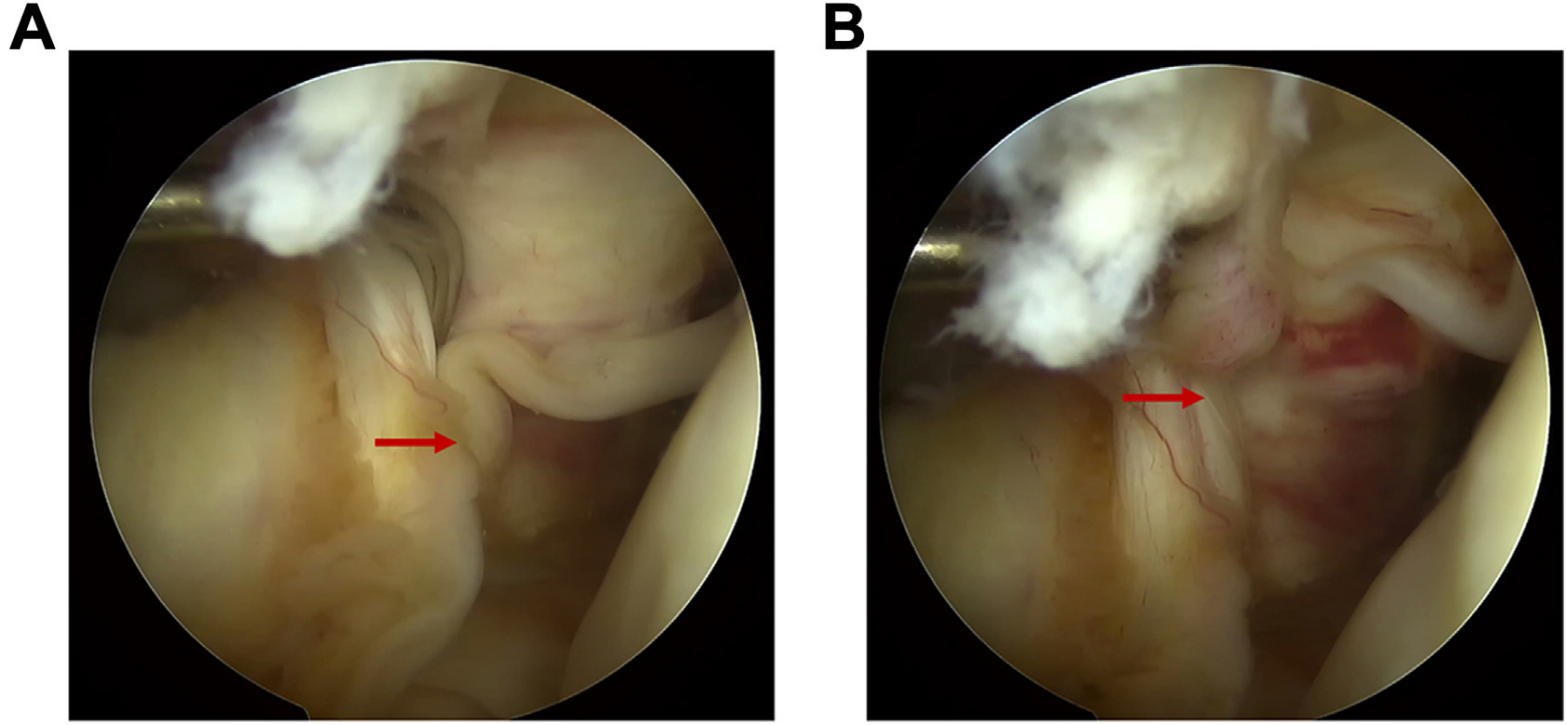
Anterior cruciate ligament rupture was observed under direct arthroscopy. This field of vision is observed through the anterolateral approach. The red arrow indicates the location of the torn anterior cruciate ligament.

An overview of our improved technique is shown in Video 1. Our enhanced technique is used in ACL reconstruction, with the same indications as general surgical guidelines for ACL reconstruction. These include definite knee instability experienced by the patient, accompanied by meniscus injury or articular cartilage injury. The patient’s anterior cruciate ligament injury needs to be confirmed by magnetic resonance imaging (MRI). This procedure necessitates the procurement and reconstruction of autograft, typically performed under general anesthesia to minimize patient discomfort. Therefore, preoperative evaluation is essential. Typically, patients who are in good health with no life-threatening comorbidities and demonstrate excellent cooperation and consent to the surgical plan are scheduled for general anesthesia. Arthroscopy is the final examination of the lesion. Figure 2 shows the anterior cruciate ligament injury in this example.

### Preparation of AperFix System-Tendon Graft-Tibial Loop Complex

**Fig 3:**
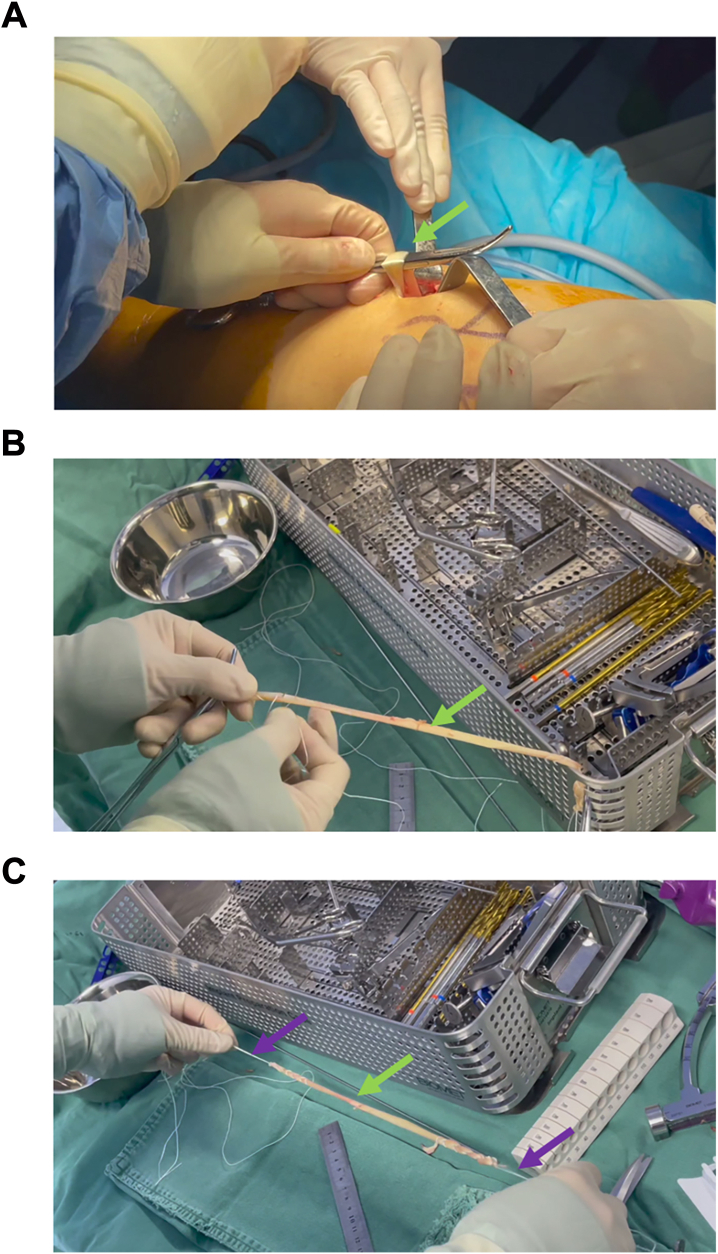
Acquisition and weaving of single semitendinosus tendon graft. (A) Harvest the semitendinosus tendon. The surgical incision was positioned medially to the tibial tubercle and measured 3 cm in length. (B and C) The 2 free ends of the tendon were sutured and braided separately. The green arrow indicates the tendon graft. The purple arrows indicate the suture used to braid the tendon.

We reconstructed the anterior cruciate ligament with single autogenous semitendinosus muscle. In contrast to the traditional all-inside technique, where both ends were used for suspensory fixation, in this technical report, the femur side was fixed by the AperFix system (Cayenne Medical, Inc., 16597 N. 92nd St., Suite 101, Scottsdale AZ), and the tibia was fixed by the traditional all-inside suspension method (ToggleLoc, Biomet Sports Medicine, Warsaw, IN). First, the tendon of the semitendinosus tendon was removed from the affected knee joint (Fig 3A). The method was to make a 3-cm oblique incision on the medial side of the tibial tubercle, expose tendon layer by layer, and take a single semitendinosus tendon. The tendon was harvested later and tiled. The two free ends of the semitendinosus tendon graft were braided and sutured (Fig 3, B and C). The length and diameter of tendon graft should be measured. In this example, the diameter of tendon graft is 8 mm, and we chose the 9-mm AperFix implant, according to the instruction.

**Fig 4:**
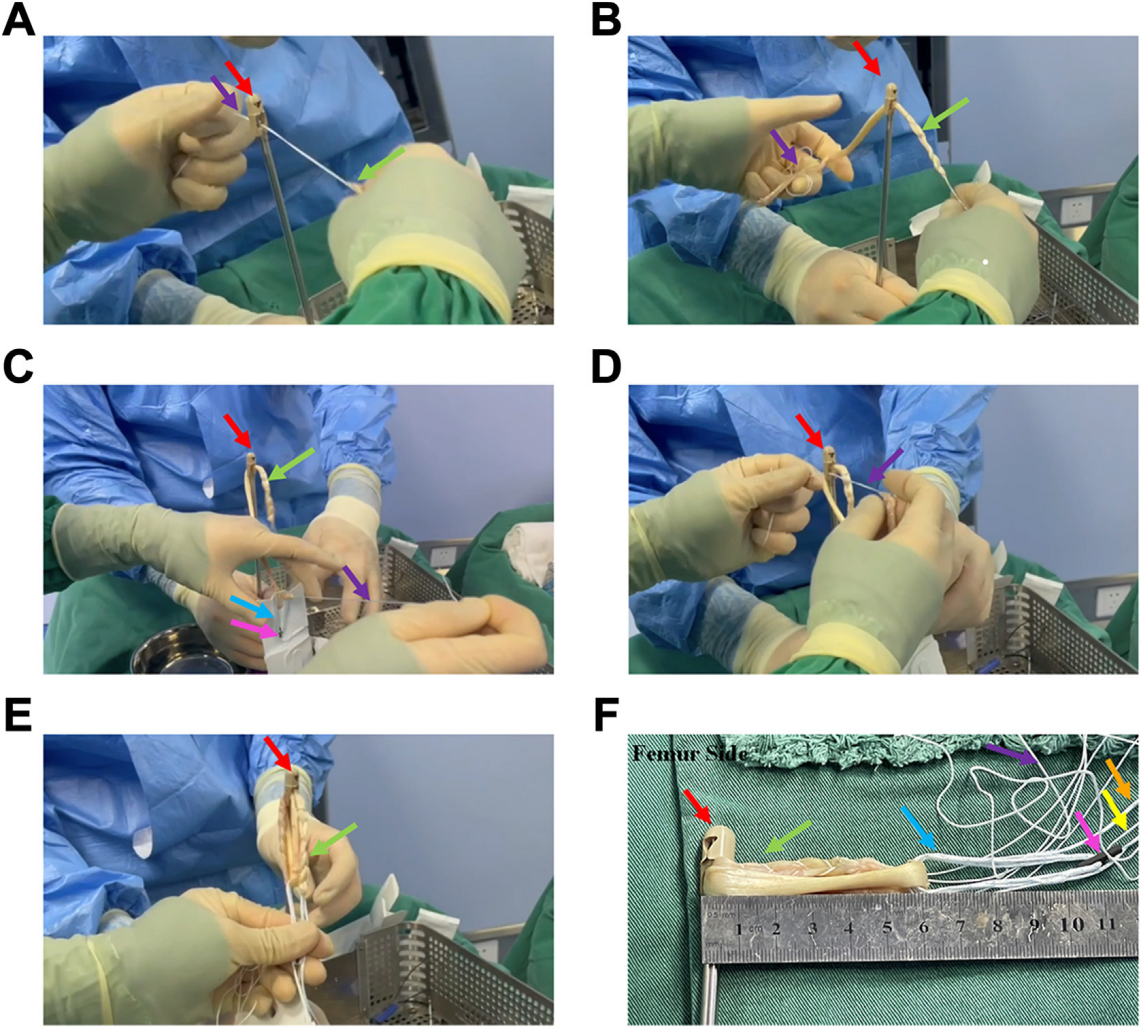
The preparation of AperFix implant + tendon graft + adjustable-loop cortical suspension devices complex. (A) Suture at one end of the graft is threaded through the AperFix implant. The red arrow indicates the AperFix implant. Purple arrows indicate the suture. The green arrow indicates the tendon graft. (B) Graft is threaded through the AperFix system. (C) The suture go through the cortical suspension device. The blue arrow indicates the adjustable loop of the cortical suspension device. The pink arrow indicates the button. (D) The tendon graft passes through the AperFix system a second time. (E and F) General view and detailed presentation of the formed complex. The yellow arrow represents the suture for pulling the button. The orange arrows represent the suture used to tighten the loop.

Then, we made a combination of AperFix implant System, Tendon Graft, and Tibial Loop Complex. The thinner end was first threaded through 1 of the 2 holes of the AperFix implant system(Fig 4A). Ensure that the length of the graft passing through the AperFix system accounts for 75% of the total length (Fig 4B). The braid suture of the graft that passes through the AperFix system was then passed through the loop of cortical suspension device (Fig 4C). Once through, thread this bundle of braided suture again through the AperFix implant system (Fig 4D). In the threading process, the length of each segment of the graft is ensured to be 1/4 of the total length. The final composite is initially formed (Fig 4E). The formed AperFix system + tendon graft + adjustable-loop cortical suspension device was formed (Fig 4F).

### The Loop-Shape Tendon Graft Complex Fixed by Knotting

**Fig 5:**
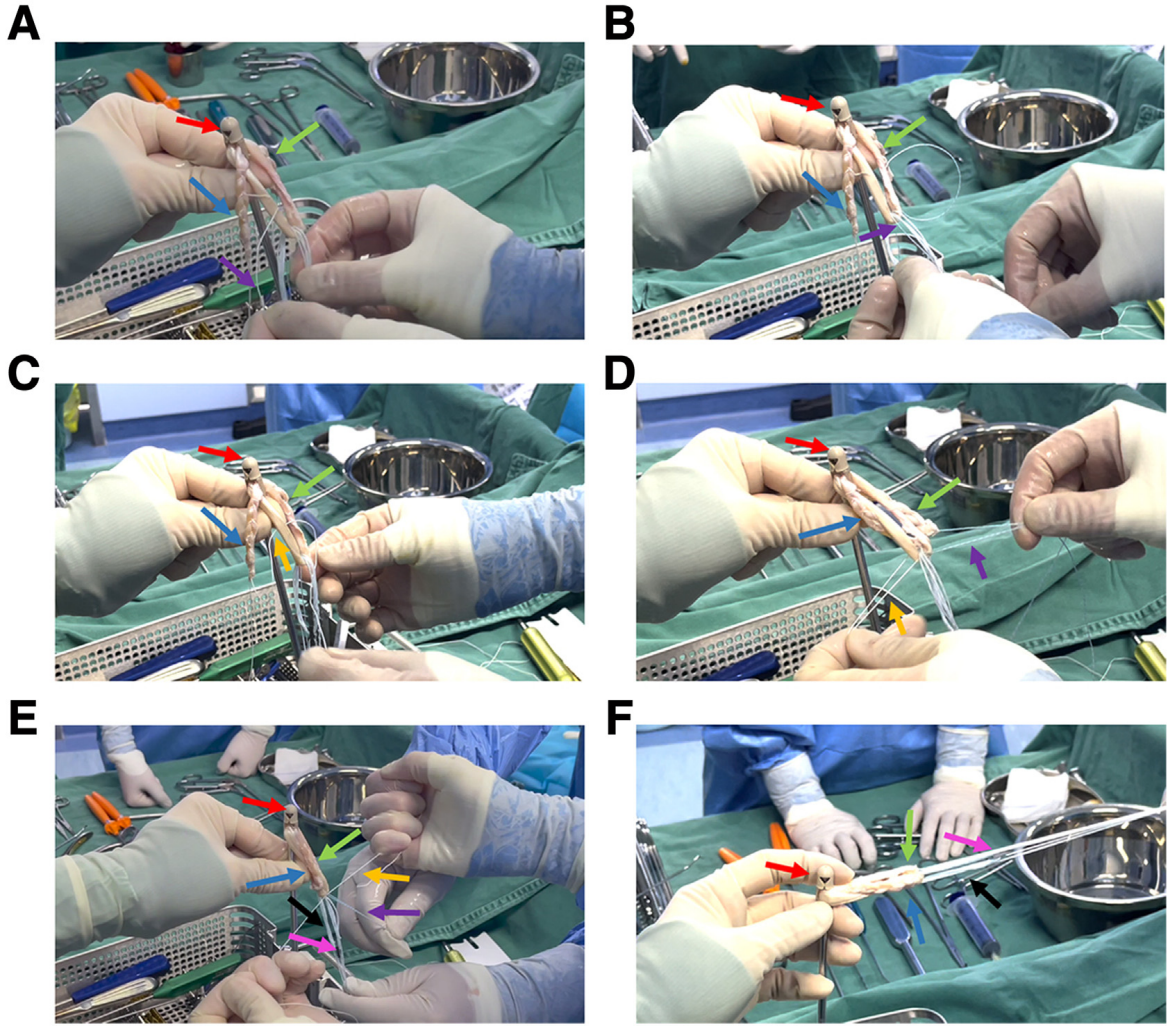
The loop-shape tendon graft complex fixed by knotting. (A) One of the sutures at one of the free ends of the semitendinosus graft is threaded through the loop of the tendon graft. The red arrow indicates the AperFix implant. Purple arrows indicate the suture of one of the free ends. The green arrow indicates one of the free ends of the semitendinosus graft. The blue arrow indicates the other free end of the semitendinosus graft. (B) Suture is then immediately threaded through adjustable-loop cortical suspension devices. (C) One of the sutures at the other free end of the graft passes through the loop of the tendon graft and immediately passes through the adjustable loop cortical suspension devices. Orange arrows indicate the suture of the other free end of the semitendinosus graft. (D) The structure of the complex before it is ready to be knotted and fixed. (E and F) The two sutures at each free end of the graft are knotted and fixed in pairs, and the graft is prepared to form a loop structure required for the all-inside technique. The black arrow indicates the adjustable loop, and the pink arrow indicates the button.

Then we performed knot fixation of tendon graft ring structure. First, one of the sutures at one of the free ends of the semitendinosus graft was threaded through the loop of the tendon graft (Fig 5A). The suture was then immediately passed through adjustable loop cortical suspension devices (Figure 5B). Similarly, one of the sutures on the other free end of the graft passes through the loop of the tendon graft and immediately passes through the adjustable-loop cortical suspension devices (Fig 5C). The formed structure is shown in Fig 4D. Finally, the two sutures at each free end of the graft are knotted and fixed in pairs, and the graft is prepared to form a circular structure required by the total internal technology (Fig 5, E and F).

### Bone Sockets Preparation

**Fig 6:**
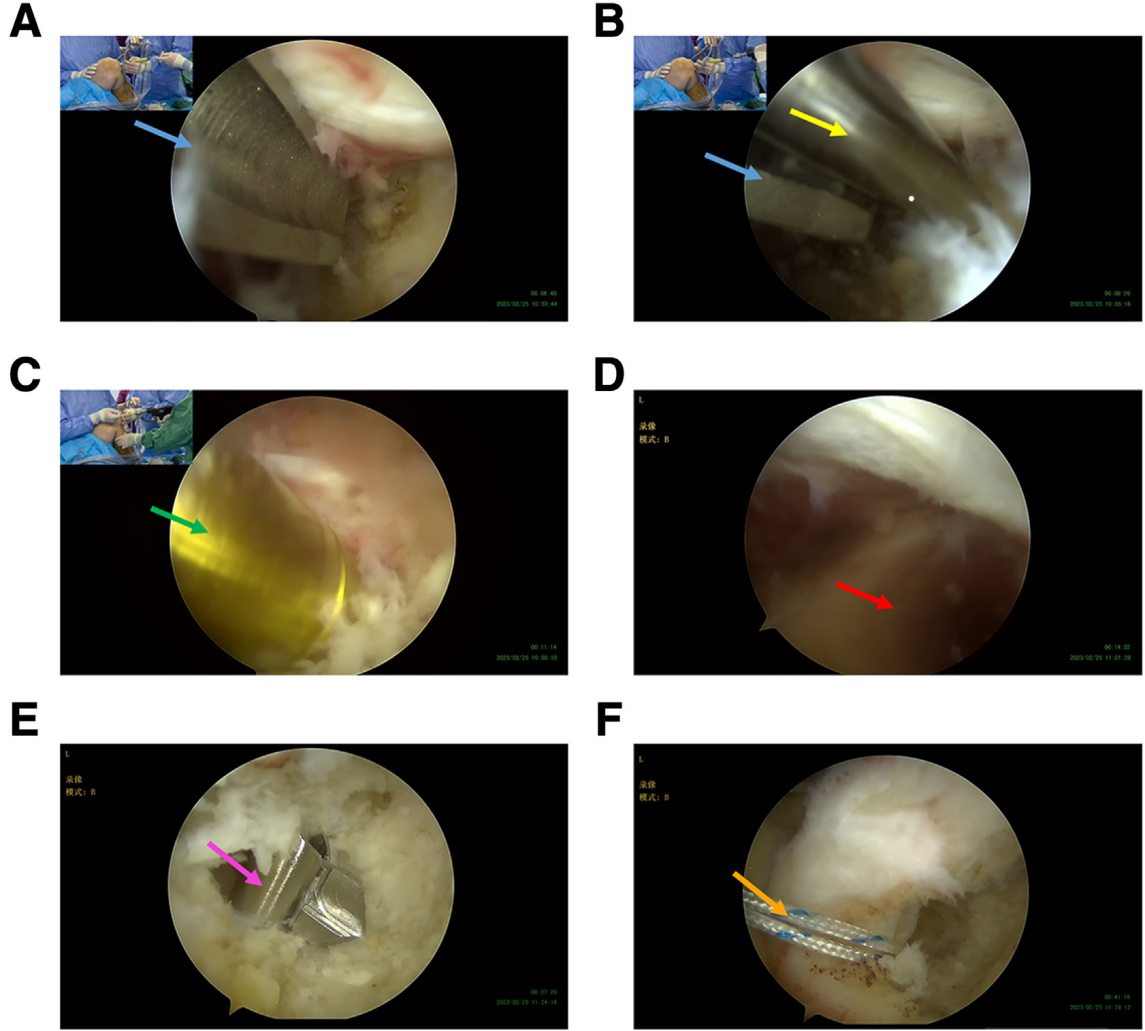
Establishment of bone sockets. The anterolateral approach is used as the observation approach, and the anteromedial approach is used as the operational approach. (A) Determination of position by the offset positioner. (B) Through the offset positioner, the 2-mm guide needle is drilled to the lateral femur cortex. This step does not require penetrating the lateral cortex of the femur. (C) Using a 9-mm femur drill bit, the surgeon prepares a 28-mm deep bone socket at the site where the guide needle was drilled. (D) The socket was fully exposed after being cleaned with a radiofrequency. The blue arrows indicate the offset positioner. The yellow arrow indicates the guide needle. The green arrow indicates the 9-mm femur drill bit. The red arrow indicates the femoral socket. (E and F) Establishment of lateral tibial bone socket. The tibial socket was achieved using the 8-mm retrograde drill (pink arrow). A traction suture (orange arrow) was inserted through the tibial socket to the intra-articular to anteromedial approach.

First, the true femoral socket was prepared. We then proceeded to immobilize the femur side of the complex. After positioning with an offset positioner(6.5 mm), a 2-mm guide needle was drilled, until it approached the cortex (Fig 6, A and B). Then a 28-mm deep bone socket could be prepared using a 9-mm femur drill bit (Fig 6C), and finally, bone and soft tissue debris could be cleaned to fully expose the prepared femoral socket (Fig 5D). Then the tibial socket was prepared. The preparation of the tibial socket is similar to the method in traditional all-inside technique. In this case, an 8-mm drill bit was used to prepare a 25-mm deep tibial socket, and a traction suture was inserted through the tibial socket to the intra-articular to anteromedial approach (Fig 6, E and F).

### Fixation of the Femur Side of the Complex

**Fig 7:**
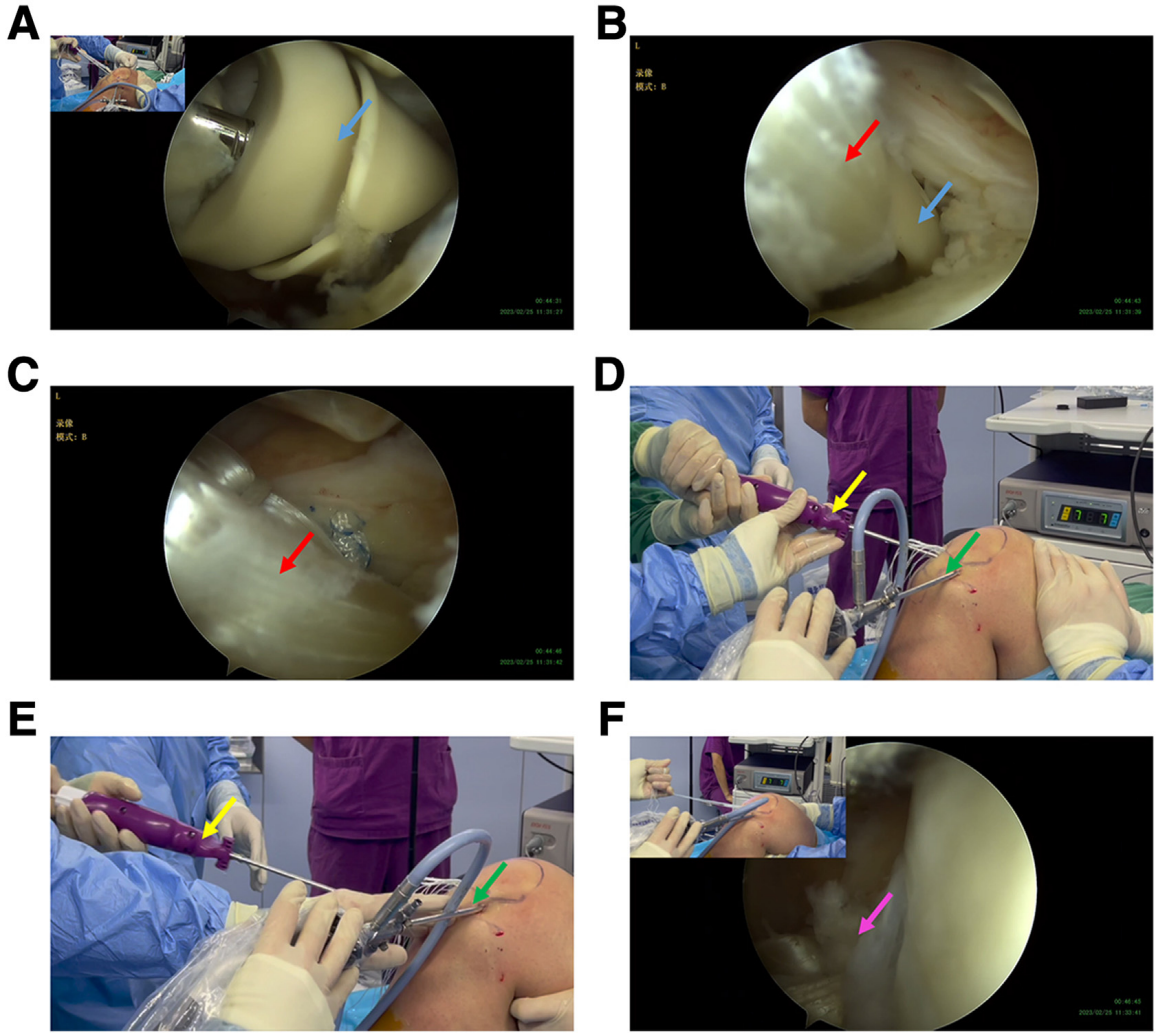
Fixation of femoral end with autogenous tendon graft complex. The anterolateral approach (green arrow) is used as the observation approach, and the anteromedial approach is used as the operational approach. (A-C) The complex is delivered into the joint cavity through the anteromedial approach, and the AperFix implant (blue arrow) is positioned into the femoral socket. AperFix implant should be fully inserted into the femoral socket and reach the bottom as far as possible. The tendon length located in the femoral socket should be maintained at 15 mm. The red arrow indicates the tendon graft. (D and E) Expand and squeeze the fixed wing of the implant through AperFix handle (yellow arrow) to secure and pull out the auxiliary device. (F) After the AperFix system is fixed, the ligament position was observed arthroscopically, and the ligament stability at femur side was tested.

The AperFix graft suspension device composite structure was implanted into the femoral socket through the anteromedial approach under direct arthroscopic vision. The entire AperFix implant body should be fully inserted into the socket and reach the socket bottom, as far as possible (Fig 7, A-C). The tendon length in the socket was set to be about 15 mm. We then activated the AperFix implant system by deploying and squeezing the fixed wing of the handle expansion screw and pulling out the auxiliary device (Fig 7, D-E). The firmness of the femoral end fixation was tested by a drawer test after the traction line was tensioned (Fig 7F).

### Fixation of the Tibia Side of the Complex

**Fig 8:**
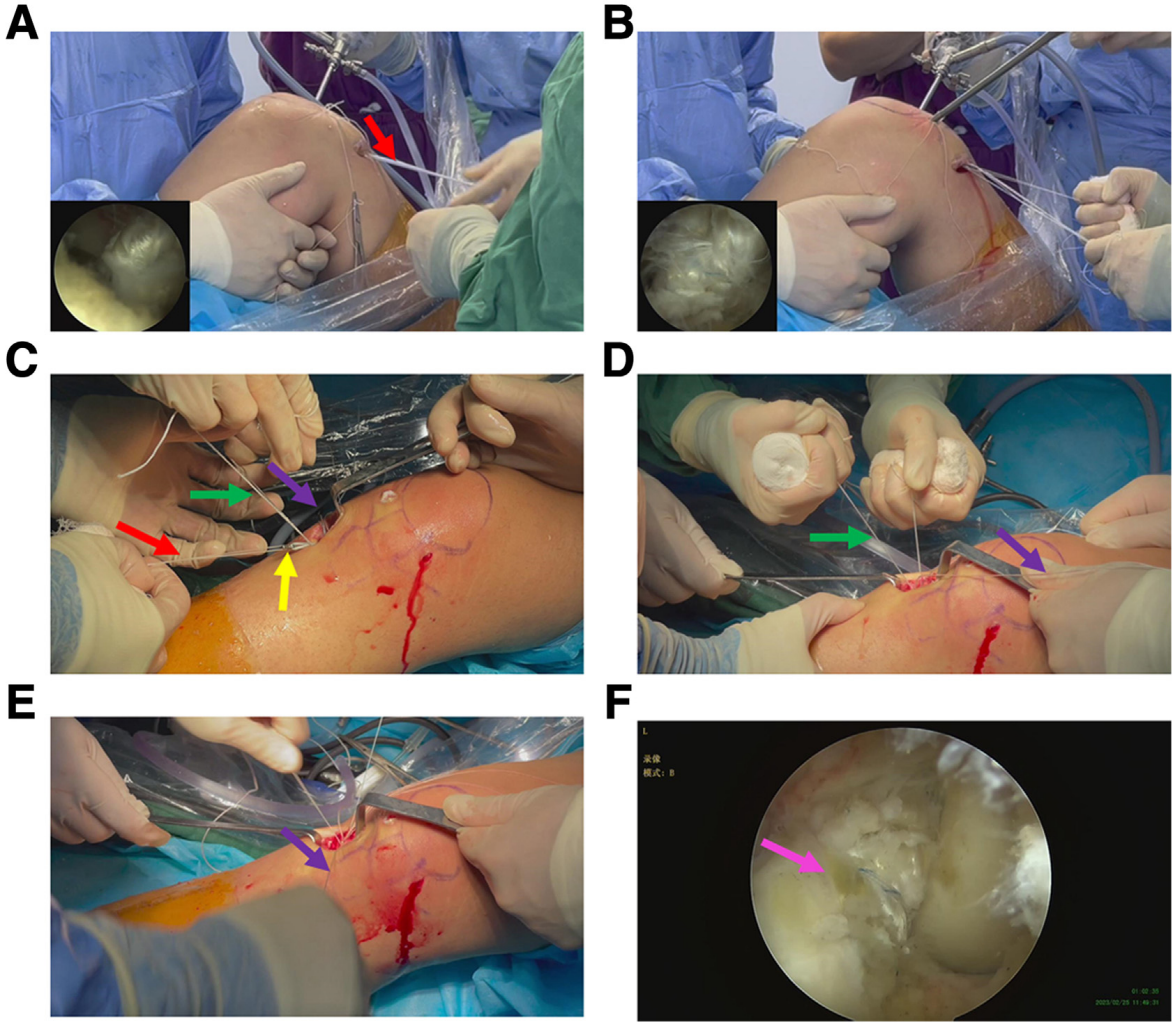
Fixation of tibial end with autogenous tendon graft complex. (A and B) Assistant tensions the braided suture and pulls the graft into the tibial socket under the direct vision of the arthroscopy. The anterolateral approach is used as the observation approach. (C-E) Tighten the adjustable loop, knot the suture, and fix the tibial cortical suspensory fixation button after 20 times of flexion and extension. The purple arrow represents the braided suture at the end of the tendon graft. The green arrows represent the tightened suture of the adjustable-loop. The red arrows represent the traction suture of the button. The yellow arrow indicates the button. (F) Examine the graft location using the anteromedial approach. The pink arrow represents the graft.

The assistant tensioned the traction suture and pulled the graft into the tibial socket under the direct vision of the arthroscopy (Fig 8, A and B). The adjustable button was then tightened after 20 flexion and extension exercises for matching (Fig 8, C-E). Finally, the sutures of the tendon on tibial side were knotted and fixed. Finally, the graft was well located by direct examination under arthroscopy (Fig 8F), the drawer test was negative, and the operation was successfully concluded.

## Discussion

ACLR is one of the most important knee arthroscopy operation. With the increasing attention to sports, more and more patients suffer from anterior cruciate ligament injury. Patients with ACL injury usually need ACLR because of knee instability. ACLR’s approach is to replace the anterior cruciate ligament with autologous, allogeneic, or artificial tendons to maintain knee stability. In conventional surgery, a bone tunnel is required in order for the tendon to be delivered and secured to the anterior cruciate ligament.^7^ However, there are many side effects associated with the presence of bone tunnels, such as tunnel expansion, local microfracture risk, bleeding, and pain. In addition, tendon grafts are long and elastic, while in traditional methods, the grafts are only fixed at 2 ends and lack intra-articular fixation, which makes the tendon grafts have tiny movements. This movement effect could cause the “windshield wiper” and “bungee cord” effects and greatly increases the ACLR’s increased risk of failure. The all-inside technique for ACLR is an important breakthrough, as this surgical procedure, for the first time, reduces the use of traditional bone tunnels and introduces the socket. However, this technology still has some shortcomings, which is reflected in the fact that the “socket” here is not a single exit tunnel in the real sense, and both ends of the socket are connected, no matter the socket on the femur side or the tibia side. As a result, patients may still have local microfracture risk and a certain risk of bleeding and pain after surgery. And because the two-end-suspension method is still used, the length of the graft has not significantly reduced the risk of “windshield wiper” and “bungee cord” effects. On the basis of these conditions, we hope that the real socket can be realized as much as possible. In this study, we implemented the femur-side femoral socket. The key to surgery is the correct application of the AperFix system. The pearls and pitfalls of surgical techniques are shown in Table 1. It is important to note that our improved technology uses a new tendon graft fixation method, which reduces the length of the graft, resulting in a significant reduction in the risk of “windshield wiper” and “bungee cord” effects. Also, our improved technique can effectively reduce the risk of postoperative tunnel expansion, pain, and bleeding in patients with less trauma. At the same time, the operation is more convenient for the surgeon, and the operation time is significantly shortened. Our improved technology has many advantages, but it also has some disadvantages, as shown in Table 2. In particular, because of technical limitations of the equipment, there is no solution that can simultaneously avoid the traditional all-inside technique, two-exit-socket of the tibia. In the future clinical practice and equipment development, the improvement of tibial fixation method of graft will be the key direction.

**Table 1:** The Pearls and Pitfalls of Improved All-Inside ACLR Using the AperFix Fixation System

Pearls	Pitfalls
1. After tendon removal, the diameter and length of the 4-strand semitendinosus grafts should be measured directly, and correct AperFix implant should be selected. Generally, AperFix implant with a diameter of 9 mm should be selected when the graft diameter is less than 8 mm or equal to 8 mm, and AperFix implant with a diameter of 10 mm should be selected for those grafts with a diameter of more than 8 mm.	1. Incorrect orientation of the spatted wings of the AperFix fixation system into the femoral socket toward the weakest posterior wall may lead to failure of fixation at the femoral end.
2. A free end of the single semitendinosus tendon is passed successively through the AperFix fixation system, the loop of the button, and again through the AperFix fixation system to form a circular all-inside ligament graft, which is generally recommended to be pierced with the thinner end of the proximal end.	2. If the posterior wall is ruptured, it may not be possible to use this femoral-side fixation technique.
3. The 2 free ends of each braided tendon at the tibial tip should be knotted on the tendon ring structure and the button loop at the same time. Finally, the 2 free ends of the braided tendon should be knotted again on the button for fixation, so as to reduce the possibility of deformation and elongation of the annular graft.	
4. The anteromedial approach technique was used to enter the femoral end socket instead of using a retrograde drill.	
5. The posterior wall of the femoral end socket should be enough thick, usually at least 2 mm. Femoral offset positioner is recommended.	
6. The orientation of the sputtered wing of the AperFix fixation system into the femoral socket should be strictly in accordance with the product instructions.	
7. Femoral-side stability of graft should be fully tested before pulling the tendon graft into the tibial socket.

**Table 2:** The Advantages and Disadvantages of Improved All-Inside ACLR Using the AperFix Fixation System

Advantages	Disadvantages
1. On the femur side, the lateral cortical bone does not need to be pierced, which is more in line with the all-inside concept and truly realizes the femoral socket technique on the femur side.	1. There is metal in the femoral socket, which may affect the postoperative imaging examination.
2. The fixation of the graft at the femur side is firmer and easier.	2. The tibial-side femoral socket of traditional all-inside technology still exists.
3. The femur side fixation is closer within the joint and reduces “windshield wiper” and “bungee cord” effects compared to traditional suspensory fixation.	

## Supplementary Data

ICMJE author disclosure forms

Video 1 Introduction of improved all-inside ACLR using the AperFix fixation system.
